# A New Surgical Technique for Ingrown Toenail

**DOI:** 10.5402/2012/438915

**Published:** 2012-05-13

**Authors:** Seyed Reza Mousavi, Jaledin Khoshnevice

**Affiliations:** Vascular Surgery & Reconstructive Cancer Research Center, Shohada—Tajrish Medical Center, Shahid Beheshti Medical Sciences, Tehran 1989934148, Iran

## Abstract

*Background*. Ingrowing toenails are a common condition which, when recurrent and painful, are often treated surgically. The aim of this study is to present a new simple surgical technique for ingrown toenails with good results. *Method and Patients*. The selected 250 patients with affected toes were surgically treated by our technique and observed from 1998 to 2004. Marginal nail elevation combined with surgical excision of the granulation tissue was more successful. For fixing the nail margin on the toe we have done one-bite suture by Nylon 3/0 that was removed after 3 weeks. *Results*. All patients were operated on by our new technique and the outcome was excellent; recurrence and failure of the technique were very low. *Discussion*. Because with this simple technique we excise the granulation tissue and elevate margin of nail over the skin, we will have higher cure rate, shorter postoperative pain, lower risk of postoperative infection, and remarkable cosmetic result without deformity; hence this technique should be considered as an alternative method of treatment.

## 1. Introduction

**Figure 1:**
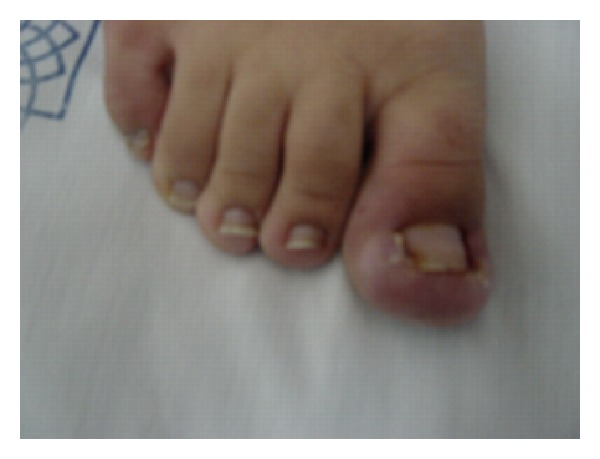
The disorder generally occurs in the great toes.

Ingrown nails, or onychocryptosis, are a common problem encountered in primary care practice [1–3] which, when recurrent and painful, are often treated surgically. The disorder generally occurs in the great toes (Figure 1), although, rarely, fingernails are involved after trauma [1]. Patients with an ingrown toenail are often in their second or third decade of life. Initially, most patients complain of pain; later, drainage and infection develop, and the patient may have difficulty walking [1, 4].

Many causes of ingrown toenail have been proposed. Two main causes are tight shoes and an incorrect nail-trimming technique [1, 5]. Shoes that fit properly may reduce the pressure between the nail and the lateral nail fold [6]. Proper trimming technique allows the corner of the nail to project beyond the edge of the skin [1]. Patients should be discouraged from tearing off the ends of the toenails, which can have the same effect as an improper trimming technique.

The numerous methods used for treating ingrowing toe-nails are testimony to the lack of a generally acceptable procedure with a low failure rate. A simple procedure with nail preservation is described, and the results of treatment are assessed.

## 2. Material and Method

At our institution, 250 patients with 285 affected toes were surgically treated by our techniques and observed form 1998 to 2009. Plastic nail wall reconstruction was effective for mild disease. Marginal nail elevation combined with surgical excision of the granulation tissue was more successful: for severe nail deformity, nail reconstruction without matrix excision was done and then our technique in the same stage was used; only one nail required additional surgical treatment. For fixing the nail margin on the toe we have done one-bite suture by Nylon 3/0 that was removed after 3 weeks.

Studies must have a minimum follow-up period of six months and aim to not remove permanently the portion of the nail.

## 3. Surgical Technique

A new simple surgical technique for ingrown toenails consisting of a resection A slice of soft tissue at the fold of the paronychium where the toenail corner enters the soft tissue is described. Before the begining of the study we received ethics approval from the ethical and law center of the Shahid Beheshti University of Medical Sciences. The procedure consists of making a transposition flap of the nail wall after preliminary excision or curettage of the granulation tissue in the nail groove.

**Figure 2:**
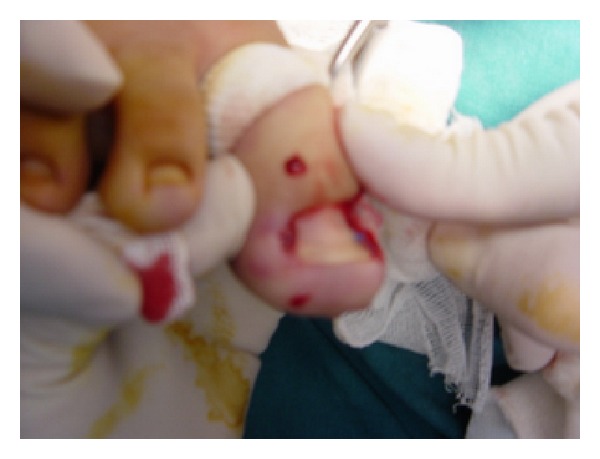
Local successful anesthesia was done.

The patients were put in supine position under local anesthesia. The local method may decrease the risk of neurovascular compromise and requires the use of less anesthetic [7]. Bilateral injection and waiting long enough for the anesthetic to have an effect are necessary for successful anesthesia with either technique.  Figure 2 and requires the use of less a minor tourniquet (with a elastic catheter) was fastened around the highest part of the toe (if possible). The foot of the affected toe was prepared and draped, so the whole foot was exposed.

**Figure 3:**
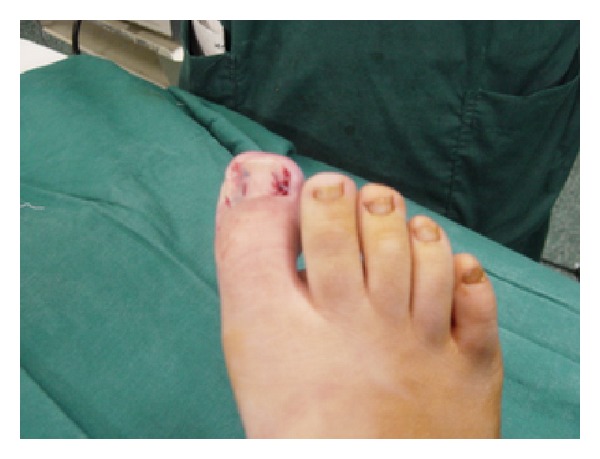
For fixing the nail margin on the toe we have done one-bite suture by Nylon.

**Figure 4:**
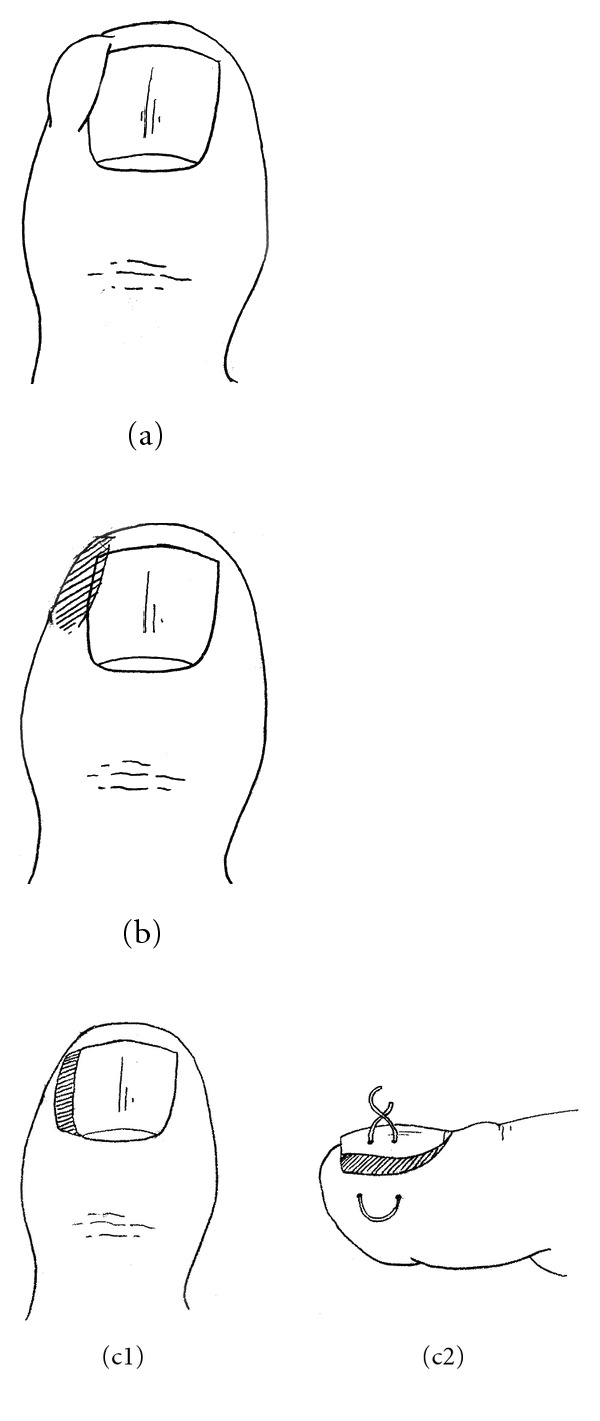
Step by step on our technique.

When cutting through the slice of soft tissue at the fold of the paronychium, the cutting instrument should not damage the overlying proximal nail fold. We prefer to bluntly separate and elevate the nail margin from the gutter using a simple nail margin elevator. After excision of the lateral granulation tissue, a flattened or crater-shaped wound may remain on the side of the toe. Adequate hemostasis should be ensured before the procedure is terminated. Lifting the lateral nail plate and try for reduce trauma to the nail bed. For fixing the nail margin on the toe we have done one-bite suture by Nylon 3/0 (Figures 3 and 4). 

After tourniquet deflation, the redundant nail from the flap edges must not be resected. We continued antibiotic therapy for three days. Three weeks later, the suture is removed.

Antibiotic ointment should be applied to the area several times a day to promote healing and to prevent infection of the burned tissues. Patients can be sent home wearing a disposable surgical slipper.

## 4. Results

Related to three stages of ingrown toenail (Table 1), we have 10 patients with 22 affected toes in stage one, 38 pateints with 51 affected toes in stage two and 102 pateints with 112 affected toes in stage three. Recurrence occurred in only 1/08% (2 of 185 toenails), and only one toe required further surgical treatment. After a maximum of six-month follow-up all of the patients were satisfied and toenails were normal. Failure of the technique in overall is 1/7%, and successful surgical treatment is over 98%.

## 5. Discussion

In the early 20th century, treatment was guided by this philosophy, “the more radical the surgery, the greater the success [2].” Recently, the value of more conservative approaches has been recognized, especially in patients with stage 1 disease [2, 5, 6, 8, 9]. Today, the most popular conservative therapies include warm water soaks, topical or oral antibiotic therapy, proper nail trimming, and elevation of the corner of the nail with a cotton wick [6, 9]. Soaking the toe in warm water for 15 minutes can reduce inflammation [6]. While antibiotics reduce bacterial infection [5], their value remains unproved in the treatment of ingrown toenails. Many treatment modalities of ingrown toenail are reported in the literature, often associated with unacceptably high recurrence rate [10]. Stage 2 disease can be managed conservatively or surgically. Some surgeons may choose to perform toenail surgery with the use of a tourniquet. However, electrosurgery may require a dry field. We use a tourniquet to provide adequate hemostasis immediately before surgery.

Surgery of the toenail should preserve as much uninvolved, normal tissue as possible. Simple partial nail technique for ingrown toenails consisting of a resection of a slice of soft tissue at the fold of the paronychium where the toenail corner enters the soft tissue (without matricectomy or permanent destruction of the nail-forming tissue) has been advocated for stage 2 ingrown toenails (Figure 3). We prefer our simple technique because partial avulsion with matricectomy may the width of the nail is reduced after healing, removal of the lateral nail without matricectomy results in recurrence of ingrown nail in 70 percent of patients [2, 11, 12]. Consequently, in our study we had 1/08% (2 of 185 toenails) recurrence. The entire nail plate should not be removed unless necessary [7] because of the resulting large area of tender, exposed nail bed.

The major contraindication to matricectomy is digital ischemia from disorders such as diabetes or peripheral or collagen vascular disease [6].

In children, conservative treatments with antimicrobial ointments, gutter treatment, and in selected cases systemic antibiotics are more promising than in adults. If these efforts remain unsuccessful, the only reliable surgical approach is a radical wedge resection [13]. Currently there are various surgical treatment modalities for ingrowing nail. None of these procedures are perfect to achieve esthetic results with low cost, recurrence, and complication rates [14].

Excision of a slice of soft tissue at the fold of the paronychium, combined with lateral nail margin elevation, suture, and fixed nail, produces excellent cure rates in patients with stage 2 and 3 disease. These techniques minimize the trauma and removal of normal tissue. This simple technique, when performed correctly, in future repositions the lateral nail groove against the cut edge of the nail.

Some physicians advocate surgical excision of the matrix [2, 6, 12]. However, extensive surgeries such as the Winograd, Zadik, or Syme procedures are time-consuming and require the placement and removal of sutures [2, 6]. Periosteal and lateral nail bed elevation procedures yield good results and maintain nail width, [15] but their use is limited because of the complex surgical technique required.

When treating a patient with a stage 3 ingrown toenail, some surgeons prefer also to surgically remove or ablate the lateral wall hypertrophy and granulation tissue [2, 8]. We suggest that the use of our technique with the removal of the lateral wall hypertrophy promotes normalization of the lateral nail fold. In addition to the high cure rate, short postoperative pain duration, and morbidity as well as low risk of postoperative infection, the remarkable esthetic results achievable with this method are indicated. As regards more definitive surgery, the simple surgical technique for ingrown toenails consisting of a resection of a slice of soft tissue at the fold of the paronychium was found to give the best results. Sepsis at the time of operation neither increased the recurrence rate nor caused severe postoperative sepsis. The treatment is effective, well tolerated, not technically difficult, of good cosmetic results, and should be considered as an alternative to current methods of treatment.

## Figures and Tables

**Table 1:** Stages of ingrown nails.

Stage	Signs and symptoms	Pateints	Affected toes
1	Erythema, slight edema, and pain when pressure is applied to the lateral nail fold	20 (8%)	32 (11/2%)
2	Increased stage 1 symptoms, drainage, and infection	78 (31/2%)	80 (28/8%)
3	Magnified stage 1 symptoms, presence of granulation tissue, and lateral wall hypertrophy	152 (60/8%)	173 (60%)
